# Association of High-Resolution Manometry Metrics with the Symptoms of Achalasia and the Symptomatic Outcomes of Peroral Esophageal Myotomy

**DOI:** 10.1371/journal.pone.0139385

**Published:** 2015-09-30

**Authors:** Yurong Tang, Chen Xie, Meifeng Wang, Liuqin Jiang, Ruihua Shi, Lin Lin

**Affiliations:** 1 Department of Gastroenterology, the First Affiliated Hospital of Nanjing Medical University, Nanjing, Jiangsu Province, China; 2 Department of Gastroenterology, Zhongda Hospital of Southeast University, NanJing, JiangSu Province, China; University Hospital Llandough, UNITED KINGDOM

## Abstract

**Background:**

High-resolution manometry (HRM) has improved the accuracy of manometry in detecting achalasia and has helped distinguish between clinically relevant subtypes. This study investigated whether HRM metrics correlate with the achalasia symptoms and symptomatic outcomes of peroral esophageal myotomy (POEM).

**Methods:**

Of the 30 patients who were enrolled, 25 were treated with POEM, 12 of who underwent HRM after 3 months. All the patients completed the Eckardt score questionnaires, and those who underwent POEM were followed up for about 6 months. Pearson correlation was used to assess the relationship between the HRM metrics and symptoms and outcomes.

**Key results:**

The integrated relaxation pressure (IRP) score positively correlated with the total Eckardt score, regurgitation score and weight loss score in all the patients, and with the weight loss score in type I achalasia. In 25 patients (10 patients, type I; 15 patients, type II) who underwent POEM, the total Eckardt scores and individual symptom scores significantly decreased after surgery. Changes in the Eckardt scores were similar between type I and type II. Further, the Eckardt scores and weight loss score changes were positively correlated with baseline IRP. Twelve patients (4 patients, type I; 8 patients, type II) underwent HRM again after POEM. IRP changed significantly after POEM, as did the DEP in type II. The IRP changes after POEM were positively correlated with the Eckardt score changes.

**Conclusions & Inferences:**

IRP is correlated with the symptoms and outcomes of achalasia patients. Thus, HRM is effective for assessing the severity of achalasia and can predict the efficacy of POEM.

## Introduction

Achalasia is an esophageal motility disorder characterized by failure of both esophagogastric junction (EGJ) relaxation and esophageal body peristalsis in response to swallowing [1]. The most common symptoms are dysphagia, regurgitation, chest pain, and weight loss [2]. The Eckardt score was developed based on these main symptoms to assess the symptomatic severity of achalasia, and is widely used in clinical practice now [3–6]. Pneumatic balloon dilatation and laparoscopic myotomy are effective treatments for achalasia. Both treatments destroy the circular muscle of the lower esophageal sphincter and result in EGJ relaxation [7, 8]. Peroral esophageal myotomy (POEM) is a novel endoscopic treatment for achalasia; it involves performing a longitudinal myotomy across the EGJ [9]. In recent years, many studies have reported excellent outcomes after POEM in terms of both symptom resolution and improvement in EGJ physiology and esophageal emptying [10, 11].

Esophageal manometry is regarded as the gold standard in the diagnosis of achalasia: it reveals peristalsis and failure of relaxation of the lower esophageal sphincter (LES) [12]. High-resolution manometry (HRM) is an evolutionary technique for the clinical evaluation of esophageal motility. It is more sensitive, provides more detailed information, and is easier to perform than conventional manometry. With HRM, achalasia is characterized by elevated integrated relaxation pressure (IRP) and absence of peristalsis [13].

Recently, a study analyzed upright HRM metrics among 269 patients (including 72 patients who had swallowing symptoms), and found that there was no correlation between HRM metrics and the symptoms [14]. Swallowing symptoms can be caused by a variety of factors, such as upper esophageal sphincter (UES) dysfunction, as suggested by some recent studies [15,16]. Achalasia is a rare esophageal motility disorder that accounts for a small proportion of patients with swallowing symptoms [17]. It is not clear whether there is a correlation between the HRM metrics and symptoms, and between the HRM metrics and the symptomatic outcomes of POEM in achalasia, particularly in the Chinese population. If such a correlation is present, HRM may also be useful for assessing the severity of achalasia symptoms and predicting the efficacy of POEM. Therefore, the present study analyzes the correlation between HRM metrics and clinical symptoms, and between HRM metrics and the symptomatic outcomes of POEM in the different types of achalasia in a population of Chinese patients.

## Patients and Methods

### Patients

First-time outpatients who attended the Department of Gastroenterology at the First Affiliated Hospital of Nanjing Medical University between January 2013 and December 2013 were recruited. The patients who were included complained of dysphagia, regurgitation, chest pain, and weight loss; they had been diagnosed with achalasia by esophageal HRM and barium swallow, and completed questionnaires that were used to calculate their Eckardt scores at the baseline. Patients with the following conditions were excluded: age less than 18 years, presence of a structural esophageal or gastric disease, history of upper gastrointestinal tract surgery, with systemic diseases that could affect esophageal motility (i.e. scleroderma and diabetes), and consumption of prokinetic medication. This retrospective study was approved by the institutional review board of the First Affiliated Hospital of Nanjing Medical University. In addition, all identifying information about the patients was removed from our records before analyses, in order to protect patient privacy.

### Symptom evaluation

The Eckardt score was used to assess the severity of achalasia symptoms. It is based on four major achalasia symptoms, using the integral system [3, 10]. The severity of dysphagia, regurgitation and chest pain were rated as follows: 0, never; 1, occasional; 2, daily; and 3, with every meal. The severity of weight loss was rated as follows: 0, no weight loss; 1, <5 kg; 2, 5–10 kg; and 3, >10 kg. The final score was the sum of the four component scores, and ranged from 0 to 12.

### HRM

All the enrolled patients underwent HRM at the baseline, and some of the patients underwent HRM again at 3 months after POEM. HRM was conducted in the supine posture after a 6-h fast. The HRM catheter (Given Imaging, Duluth, GA) was placed transnasally in order to record the pressure from the hypopharynx to the stomach. The manometric protocol included 1-min baseline recording and 10 swallows of 5 ml of warm water. The HRM results were analyzed using the Manoview analysis software (Given Imaging) [18]. According to the Chicago Classification Criteria, an IRP value of over 15 mm Hg is indicative of achalasia, and based on esophageal body peristalsis, achalasia can be divided into three subtypes: type I, 100% failure of peristalsis; type II, no normal peristalsis and ≥20% swallowing with panesophageal pressurization; type III, no normal peristalsis and ≥20% swallowing with preserved spastic contractions [13]. The following metrics were recorded: IRP, LES resting pressure (LESP), LES length (LESL), distal esophageal pressure (DEP), panesophageal pressurization rate (PPR) in type II achalasia, and spastic contraction rate in type III achalasia.

### POEM

The POEM technique is based on the one reported by Inoue and colleagues, and has been previously described in detail [19]. It involves longitudinal myotomy of the EGJ. All the POEM procedures were performed by the same operator.

### Statistical analysis

Statistical analysis was performed using the SPSS software (version 19; IBM, NY, USA). Data are presented as mean ± standard deviation. Differences in the HRM metrics and Eckardt scores between groups were analyzed using one-way analysis of variance. Relationships between the HRM metrics and Eckardt scores were assessed using Pearson’s correlation coefficient analysis. A two-tailed p value of 0.05 was considered to indicate statistical significance in all cases.

## Results

### Patient status

A total of 30 patients with achalasia were enrolled, and all of them completed the questionnaires for Eckardt scores and underwent HRM. They were divided into the three achalasia types according to the HRM findings. Twenty-five of them underwent POEM. After the surgery, one patient was lost to follow-up, and the rest of the 24 patients were evaluated using the Eckardt score again. Twelve of the patients underwent HRM again at 3 months after POEM. Because only one patient had type III achalasia, we mainly compared patients with type I and type II achalasia. There was no difference in the gender, age and disease course between patients with the different achalasia types (Table 1).

**Table 1:** Demographic characteristics, Eckardt scores and HRM metrics of the 30 patients with achalasia at the base line.

Demographic characteristics, Eckardt scores and HRM metrics	Achalasia types				*P* value^#^
	Total patients(n = 30)	Type I(n = 13)	Type II(n = 16)	Type III(n = 1)	
Demographic characteristics					
Gender (female/male)	20/10	10/3	10/6	0/1	0.404
Age (yr)	37.3 ± 14.4	34.0 ± 12.7	38.2 ± 15.4	56	0.366
Disease course (yr)	4.2 ± 3.5	4.5 ± 3.4	3.9 ± 3.8	3	0.656
Eckardt scores					
Total Eckardt score	6.33 ± 2.25	5.69 ± 2.10	7.00 ± 2.25	4	0.120
Dysphagia	2.80 ± 0.41	2.85 ± 0.38	2.81 ± 0.40	2	0.819
Regurgitation	2.00 ± 0.95	1.54 ± 1.05	2.38 ± 0.72	2	0.017*
Chest pain	0.53 ± 0.73	0.54 ± 0.66	0.56 ± 0.81	0	0.932
Weight loss	1.00 ± 1.0	0.77 ± 1.01	1.25 ± 1.00	0	0.211
HRM metrics					
IRP (mmHg)	21.73 ± 6.49	20.09 ± 6.90	23.06 ± 6.03	15.8	0.228
LESP (mmHg)	24.26 ± 11.45	23.35 ± 13.41	25.39 ± 10.23	18.1	0.645
LESL (cm)	3.91 ± 0.78	3.95 ± 0.62	3.88 ± 0.91	3.9	0.812
DEP (mmHg)	20.80 ± 16.14	7.34 ± 6.21	31.74 ± 13.06	68.8	0.000*
PPR (%)	/	/	75.50 ± 29.60	/	/

^#^ *P* value, type I *vs*. type II.

*P < 0.05.

HRM, high-resolution manometry; IRP, integrated relaxation pressure; LESP, resting lower esophageal sphincter pressure; LESL, lower esophageal sphincter length; DEP, mean distal esophageal pressure; PPR, panesophageal pressurization rate.

### Baseline data

**Fig 1:**
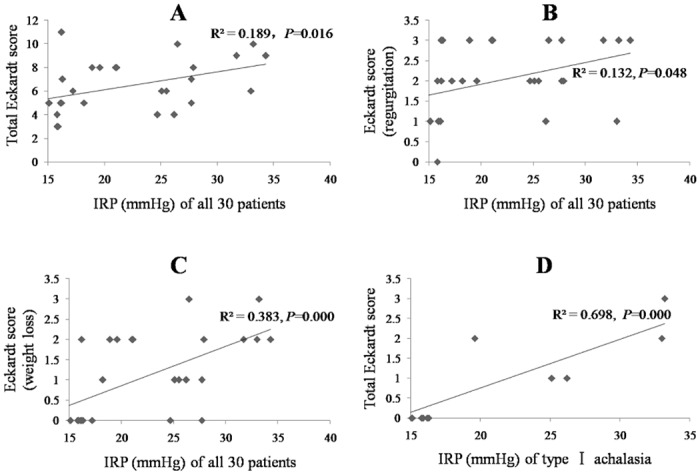
Correlation between HRM metrics and Eckardt scores at the baseline. (A) Correlation between IRP and the total Eckardt score in all 30 patients, (B) correlation between IRP and the Eckardt score for regurgitation in all 30 patients, (C) correlation between IRP and the Eckardt score for weight loss in all 30 patients, (D) correlation between IRP and the Eckardt score for weight loss in type I achalasia patients.

1. Eckardt scores at the baseline: As shown in Table 1, the Eckardt scores (total score, dysphagia, regurgitation, chest pain and weight loss) of the 30 patients, and the achalasia types were record. Because only one patient had type III achalasia, we mainly compared the Eckardt scores of patients with type I and type II. The only difference was in the regurgitation score between type I and type II achalasia (*P* = 0.017).
2. HRM metrics at the baseline: The HRM metrics (IRP, LESP, LESL, DEP and PPR) of the 30 patients and each achalasia type are shown in Table 1. The IRP, LESP and LESL values for both types (type I and type II) were similar (*P* > 0.05 for all). DEP was different between type I and type II achalasia, which is consistent with the definition for these types (*P* = 0.000). The PPR for type II achalasia was 75.50% ± 29.60%.
3. Correlation between the HRM metrics and Eckardt scores at the baseline: *P* values for the correlation between each HRM metric and Eckardt score are listed in Table 2. IRP was positively correlated with the total Eckardt score (*P* = 0.016, as shown in Fig 1A), regurgitation score (*P* = 0.048, as shown in Fig 1B) and weight loss score (*P* = 0.000, as shown in Fig 1C) in all the achalasia patients. Moreover, it was correlated with the weight loss score in type I achalasia patients (*P* = 0.000, as shown in Fig 1D). LESP was positively correlated with the weight loss score (*P* = 0.029) in all the achalasia patients. No correlation was found between the Eckardt scores and the other HRM metrics.

**Table 2:** Correlation between HRM metrics and Eckardt scores at the baseline (*P* values).

Eckardt scores		HRM metrics				
		IRP	LESP	LESL	DEP	PPR
Total (n = 30)	Total score	0.016*	0.219	0.593	/	/
	Dysphagia	0.326	0.667	0.462	/	/
	regurgitation	0.048*	0.207	0.745	/	/
	chest pain	0.610	0.337	0.819	/	/
	weight loss	0.000*	0.029*	0.675	/	/
Type I (n = 13)	Total score	0.143	0.878	0.633	0.822	/
	Dysphagia	0.842	0.853	0.552	0.440	/
	regurgitation	0.494	0.941	0.429	0.785	/
	chest pain	0.481	0.111	0.803	0.941	/
	weight loss	0.000*	0.086	0.817	0.675	/
Type II (n = 16)	Total score	0.209	0.475	0.415	0.567	0.825
	Dysphagia	0.210	0.686	0.601	0.603	0.500
	regurgitation	0.086	0.200	0.291	0.204	0.170
	chest pain	0.795	0.797	0.720	0.899	0.338
	weight loss	0.179	0.450	0.568	0.934	0.559

*P < 0.05.

HRM, high-resolution manometry; IRP, integrated relaxation pressure; LESP. Resting lower esophageal sphincter pressure; LESL, lower esophageal sphincter length; DEP, mean distal esophageal pressure; PPR, panesophageal pressurization rate.

### Changes in the Eckardt score and HRM parameters in the achalasia patients after POEM

**Fig 2:**
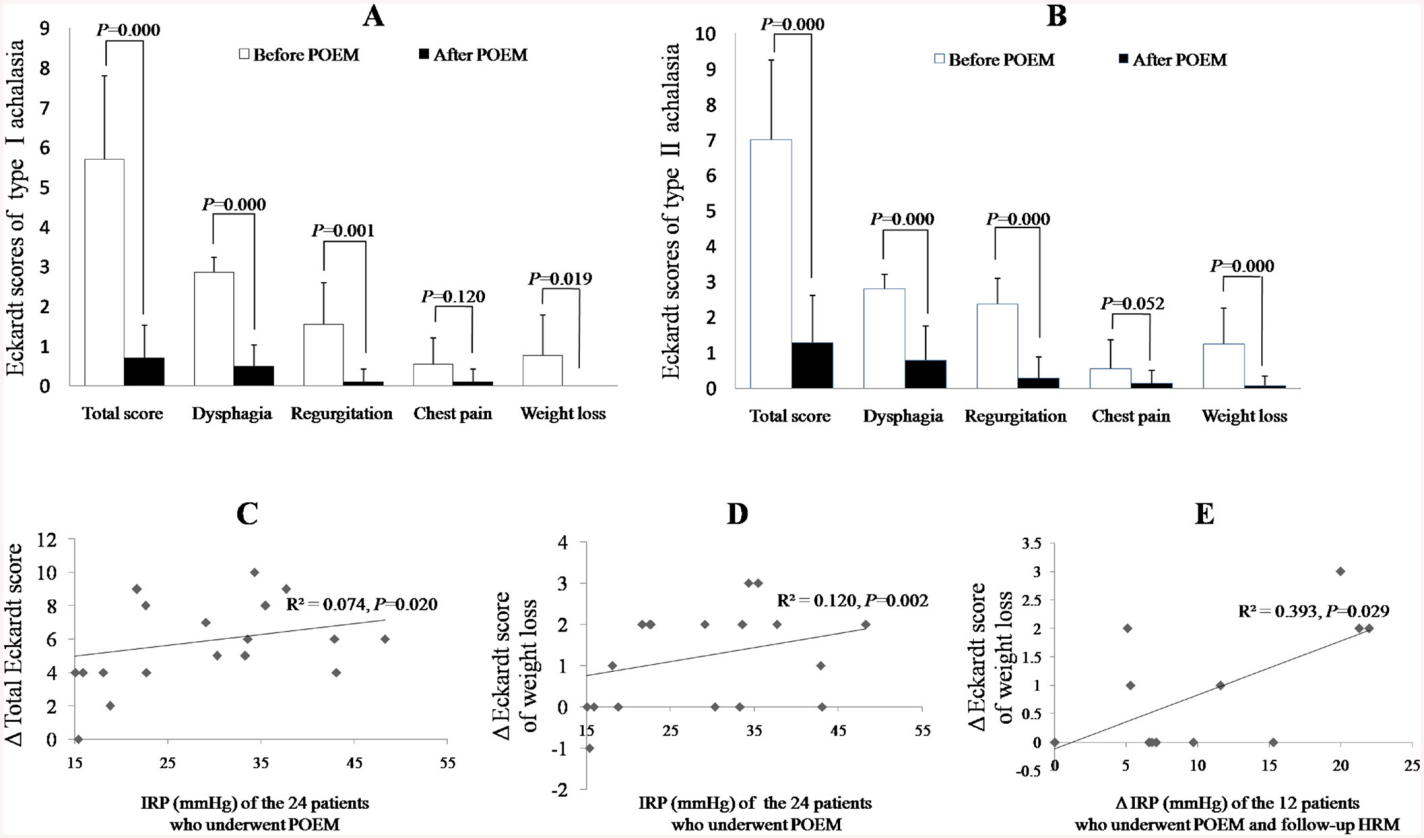
Changes in the Eckardt score of patients with achalasia after POEM and its correlation with IRP. Changes in the Eckardt score of patients with type I (A, n = 10) and type II (B, n = 14) achalasia after POEM: correlation between IRP before POEM and changes in the total Eckardt score (C), correlation between IRP before POEM and changes in the Eckardt score for weight loss after POEM (D, n = 24), and (E) correlation between IRP changes and changes in the Eckardt score for weight loss after POEM (n = 12).

1. Changes in the Eckardt score of the 24 patients with achalasia after POEM: Among all the patients enrolled, 24 (10 with type I and 14 with type II) underwent both POEM and completed the Eckardt score evaluation after POEM. As shown in Fig 2A and 2B, the total Eckardt scores and scores for each symptom decreased after surgery in both type I and type II achalasia patients (*P* < 0.05 for all); the only exception was the chest pain score, which did not show a significant decrease after POEM (*P* > 0.05 for all). The difference between the total Eckardt scores and Eckardt score changes for each symptom before POEM and after POEM (ΔEckardt scores) were similar between type I and type II achalasia (*P* > 0.05 for all).
2. Changes in the HRM metrics in the 12 patients with achalasia after POEM: Among the patients who underwent POEM, 12 (4 with type I and 8 with type II) of them underwent HRM again at 3 months after POEM. As shown in Table 3, IRP and LESP decreased in all the 12 patients (*P* = 0.005 and 0.021 respectively), and DEP in type II achalasia also decreased (*P* = 0.010). No changes in the other HRM metrics were found after POEM.

**Table 3:** Changes in the HRM metrics in the 12 patients with achalasia after POEM.

HRM metrics	After POEM	Δ(before—after)	P value(after *vs*. before)
IRP (mmHg, n = 12)	11.13 ± 3.47	10.90 ± 7.19	0.005*
LESP (mmHg, n = 12)	15.86 ± 6.48	10.06 ± 6.48	0.021*
LESL (cm, n = 12)	3.56 ± 1.11	0.06 ± 1.41	0.577
DEP (mmHg, n = 12)			
Type I (n = 4)	12.75 ± 6.75	-3.13 ± 6.59	0.456
Type II (n = 8)	12.45 ± 9.02	16.29 ± 13.43	0.010*
PPR (mmHg, type II, n = 8)	33.75 ± 33.35	41.25 ± 48.24	0.549

Δ, decrease in the value of each metric after POME.

*P < 0.05.

HRM, high-resolution manometry; IRP, integrated relaxation pressure; LESP, resting lower esophageal sphincter pressure; LESL, lower esophageal sphincter length; DEP, mean distal esophageal pressure; PPR, panesophageal pressurization rate.

### Correlation between the HRM metrics and symptomatic outcomes of POEM in achalasia

1. Correlation between the demographic characteristics/HRM metrics before POEM and changes in the Eckardt score after POEM: The *P* values of the correlation between each demographic characteristic/HRM metric before POEM and changes in the Eckardt score after POEM are listed in Table 4. IRP before POEM was positively correlated with changes in the total Eckardt score (*P* = 0.020, as shown in Fig 2C) and changes in the weight loss score (*P* = 0.002, as shown in Fig 2D) in all the achalasia patients. No correlation was found between changes in the Eckardt score and the other HRM metrics before POEM or the demographic characteristics.
2. Correlation between changes in the HRM metric and changes in the Eckardt scores after POEM:
  The *P* values of the correlation between changes in each HRM metric and changes in the Eckardt score after POEM are listed in Table 5. Only the IRP changes positively correlated with the weight loss changes (*P* = 0.020, as shown in Fig 2E) in the 12 patients.

**Table 4:** Correlation between demographic characteristics / HRM metrics and Eckardt score changes after POEM (*P* value, n = 24).

Eckardt score changes	Demographic characteristics			HRM metrics					
	Gender	Age	Course	IRP	LESP	LESL	DEP		PPR (Type II)
							Type I	Type II	
Δ total score	0.683	0.581	0.904	0.020*	0.197	0.771	0.600	0.762	0.913
Δ Dysphagia	0.302	0.259	0.652	0.388	0.285	0.765	0.226	0.733	0.728
Δ regurgitation	0.281	0.093	0.814	0.080	0.516	0.732	1.000	0.832	0.816
Δ chest pain	0.764	0.321	0.931	0.682	0.801	0.968	0.534	0.552	0.499
Δ weight loss	0.517	0.929	0.837	0.002*	0.097	0.503	0.454	0.852	0.720

Δ, decrease value of each scores after POEM.

*P<0.05.

HRM, High-resolution manometry. IRP, integrated relaxation pressure, LESP. Resting lower esophageal sphincter pressure. LESL, lower esophageal sphincter length. DEP, mean distal esophageal pressure. PPR, panesophageal pressurization rate.

**Table 5:** Correlation between changes in the HRM metrics and changes in the Eckardt score after POEM (n = 12).

Eckardt score changes	HRM metrics				
	Δ IRP	Δ LESP	Δ LESL	Δ DEP (Type II, n = 8)	Δ PPR (Type II, n = 8)
Δ total score	0.186	0.392	0.515	0.203	0.703
Δ dysphagia	0.934	0.546	0.309	0.066	0.574
Δ regurgitation	0.627	0.763	0.884	0.839	0.566
Δ chest pain	0.501	0.843	0.711	0.057	0.645
Δ weight loss	0.029*	0.185	0.979	0.844	0.869

Δ, decrease in the value of each score after POEM.

*P < 0.05.

HRM, high-resolution manometry; IRP, integrated relaxation pressure; LESP, resting lower esophageal sphincter pressure; LESL, lower esophageal sphincter length; DEP, mean distal esophageal pressure; PPR, panesophageal pressurization rate.

## Discussion

The correlation between HRM metrics and clinical outcomes and HRM metrics and the symptomatic outcomes of POEM was examined in 30 Chinese patients with type I and type II achalasia.

At the baseline, the total Eckardt score and the scores for dysphagia, chest pain and weight loss, except for the regurgitation score, were similar in both type I and type II achalasia. These findings indicate that patients with different types of achalasia may have no significant difference in their symptoms. We also found that most of the HRM metrics (IRP, LESP and LESL) were similar in both types (type I and type II), which is consistent with the symptoms observed in this study. DEP was different between type I and type II achalasia, consistent with the definition of these types. These findings imply that the type of achalasia cannot be determined according to the symptoms, but only by the findings of esophageal manometry. Some other studies have also reported that there were no significant differences in the timed barium esophagogram measurements between patient groups [18].

Numerous studies have reported that responses to treatment are closely related to the achalasia type [6, 20, 21]. Type II patients are significantly more likely to respond to most of the therapies (BoTox, pneumatic dilation, or Heller myotomy) than type I or type III patients [6, 20]. Moreover, patients with type III can probably best be treated by Heller myotomy [21]. Considering the risks of surgery, POEM is thought to be a better choice for achalasia patients and has been reported to entail lower risk in recent years [22, 23]. Further, it was reported that POEM considerably decreased the Eckardt score of achalasia patients [19, 24]. However, according to the findings of our study, POEM significantly relieved the symptoms of achalasia as reported by the patients, and the efficacy was not different between type I and type II patients. This means that the achalasia type is not sufficient for predicting the efficacy of POEM.

In this study, we found that some HRM metrics, such as IRP, were positively correlated with the total Eckardt score, regurgitation score and weight loss score in all the achalasia patients, and that it was also correlated with the weight loss score in type I achalasia. IRP represents the mean EGJ pressure over four contiguous or non-contiguous seconds of relaxation in the 10-s window following deglutitive UES relaxation. It was reported that the 4-s IRP with a cutoff of 15 mm Hg was optimal for the diagnosis of achalasia, with 98% sensitivity and 96% specificity [25]. Thus, IRP has important diagnostic value in achalasia, but it is not clear whether it is associated with the severity of the disease. In a recent study, the upright HRM metrics of 269 patients with an upright swallow symptom score of ≥1 were analyzed, but no correlation was found between the HRM metrics and symptoms [14]. However, another study demonstrated that there are variations in the IRP values when patients are in different detection postures [26]. The Chicago Classification of esophageal motility disorders is based on swallowing in the supine position. In accordance with this, our results for the correlation between IRP and symptoms were based on examination in the supine position for patients diagnosed with achalasia. Additionally, among the symptoms for which the Eckardt scores were calculated, most of them were subjective, and weight loss was more objective. We found that IRP was positively correlated with weight loss in all the achalasia patients and in type I achalasia. Therefore, we believe that the IRP value is consistent with the severity of achalasia.

Our results indicated that IRP changes were positively correlated with weight loss changes after POEM. This implies that IRP is a meaningful indicator for assessing the efficacy of POEM in achalasia. We also found that IRP before POEM was positively correlated with the total Eckardt score change and weight loss score change in all the achalasia patients. The findings also suggested that IRP could predict the efficacy of POEM, as higher IRP values predicted more obvious improvements in the symptoms after surgery. This is consistent with previously reported findings for pneumatic balloon treatment of achalasia [27].

LESP was previously considered as an objective indicator of the symptoms of achalasia and is often used for objective assessment of the severity of achalasia or therapeutic efficacy [10, 19, 28, 29]. From this study, we found that LESP was less sensitive than IRP. LESP was only correlated with weight loss in the entire cohort. Moreover, LESP decreased after POEM, but its decrease was not associated with changes in the Eckardt score. Additionally, the LESP value before POEM could not predict changes in the Eckardt score. A recent study reported that there was no association between LESP and IRP, or between the LESP value before treatment and treatment response [21]. LESP forms a part of the EGJ resting pressure, which might not be directly related to the EGJ relaxation ability during swallowing.

There are two main limitations to our study. The first limitation is the small patient population: in particular, only one of the patients had type III achalasia. A larger sample and a prospective study are required for more accurate and more meaningful data. Secondly, the HRM metrics analyzed do not include the UES functions which, according to a recent report, might be related to achalasia symptoms [16].

In summary, in this study, we found that (1) the Eckardt scores and HRM metrics were similar between type I and type II achalasia; (2) IRP was positively correlated with the Eckardt scores in achalasia patients; (3) the Eckardt scores decreased after POEM, and IRP in all the patients and DEP in type II achalasia patients changed significantly after POEM; (4) the changes in the Eckardt scores were not different between type I and type II achalasia, but were positively correlated with IRP changes and IRP at the baseline. From these findings, we can conclude that IRP is correlated with the symptoms and symptomatic outcomes of POEM in achalasia patients. Further, we can also say that HRM is an effective means of assessing the severity of achalasia, and can be used to predict the efficacy of POEM.

## Supporting Information

S1 Fig Flow chart showing study enrollment and procedures. A total of 30 patients with achalasia were enrolled, and all of them completed the questionnaires for Eckardt scores and underwent HRM. They were divided into the three achalasia types according to the HRM findings. Twenty-five of them underwent POEM. After the surgery, one patient was lost to follow-up, and the rest of the 24 patients were evaluated using the Eckardt score again. Twelve of the patients underwent HRM again at 3 months after POEM.
(TIF)
