# Supplementary figures and images for: Association of High-Resolution Manometry Metrics with the Symptoms of Achalasia and the Symptomatic Outcomes of Peroral Esophageal Myotomy
Source: PLoS One. 2015 Sep 30;10(9):e0139385. doi: 10.1371/journal.pone.0139385 (PMC4589231; doi:10.1371/journal.pone.0139385)

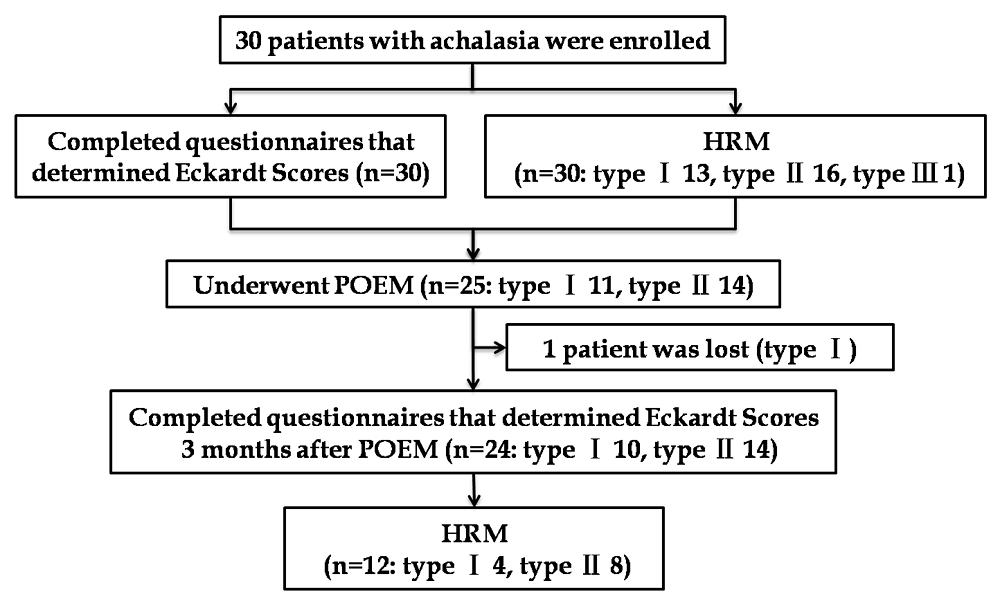

Supplement: S1 Fig — A total of 30 patients with achalasia were enrolled, and all of them completed the questionnaires for Eckardt scores and underwent HRM. They were divided into the three achalasia types according to the HRM findings. Twenty-five of them underwent POEM. After the surgery, one patient was lost to follow-up, and the rest of the 24 patients were evaluated using the Eckardt score again. Twelve of the patients underwent HRM again at 3 months after POEM. (TIF) [file pone.0139385.s001.tif]
